# Supplementary material for: The Systems Biology Research Tool: evolvable open-source software
Source: BMC Syst Biol. 2008 Jun 29;2:55. doi: 10.1186/1752-0509-2-55 (PMC2446383; doi:10.1186/1752-0509-2-55)
Supplement: Additional file 1 — SBRT Archive. An archive of the current version of the Systems Biology Research Tool. [file 1752-0509-2-55-S1.zip › sbrt-1.4.0/doc/developers_guide/api/sbrt/kernel/math/algebra/LinearSystemSolution.html]

LinearSystemSolution


|  |  |  |
| --- | --- | --- |
| **Overview** \| **Package** \| **Class** \| **Use** \| **Tree** \| **Deprecated** \| **Index** \| **Help** | |  |
| **PREV CLASS**   **NEXT CLASS** | **FRAMES**    **NO FRAMES**     **All Classes** |  |
| SUMMARY: NESTED \| FIELD \| CONSTR \| METHOD | DETAIL: FIELD \| CONSTR \| METHOD |  |


---


## sbrt.kernel.math.algebra Interface LinearSystemSolution<V>

**Type Parameters:**: `V` - the variable type.

**All Known Implementing Classes:**: HashLinearSystemSolution

---

``` public interface LinearSystemSolution<V> ```

This interface is used to represent a general solution to a
system of linear equations. It is primarily intended for underdetermined
systems, whose solutions consist of both free and fixed variables.
In this representation, the variables
contained in the
solution are the same as those contained in the original system of equations.
So in the solution, each variable from the original system is equal to a
linear combination
of those same variables. Take, for instance, the system

x + y = 1

2x + 2y = 2.

One solution is

x = x

y = 1 - x,

where x is a free variable and y is fixed. By assigning a value to x, the
value of y is also determined. Another equivalent solution is

x = 1 - y

y = y,

where this time y is a free variable and x is fixed. Note that free variables
are simply equal to themselves, and fixed variables are equal to linear
combinations of free variables. Some fixed variables may even equal just
a constant. This interface captures
these basic facts about solutions to systems of linear equations.

**Author:**
:   This interface was written and documented by
    Jeremiah Wright while in the Wagner lab.

---

| **Method Summary** | |
| --- | --- |
| `int` | `fixedVariables()`             Returns the number of fixed variables in this solution. |
| `int` | `freeVariables()`             Returns the number of free variables in this solution. |
| `java.util.Set<V>` | `getFreeVariables()`             Returns the set of free variables contained in this solution. |
| `LinearComb<V>` | `getSolution(java.lang.Object variable)`             Returns the linear combination of free variables to which the provided variable is equal. |
| `java.util.Map<V,java.lang.Double>` | `getSolutionVector(java.util.Map<?,java.lang.Double> freeVariableValues)`             Computes and returns a solution vector by plugging in the provided values for the free variables in this solution. |
| `java.util.Set<V>` | `getVariables()`             Returns the set of variables contained in this solution. |
| `boolean` | `isSolutionVector(java.util.Map<?,java.lang.Double> variableValues, double tolerance)`             Indicates if the provided solution vector is consistent with this solution. |
| `boolean` | `isVariable(java.lang.Object obj)`             Indicates if the provided object is a variable in this solution. |
| `LinearSystemSolution<V>` | `simplify(java.util.Map<?,java.lang.Double> variableValues, double tolerance)`             Computes and returns a simplified solution by plugging in the provided values. |
| `java.util.Map<V,LinearComb<V>>` | `toMap()`             Returns this solution as a map. |
| `int` | `variables()`             Returns the total number of variables in this solution. |

**Method Detail**

### getVariables

```
java.util.Set<V> getVariables()
```

:   Returns the set of variables contained in this solution.

    :   **Returns:**: the set of variables contained in this solution.

---


### getFreeVariables

```
java.util.Set<V> getFreeVariables()
```

:   Returns the set of free variables contained in this
    solution.

    :   **Returns:**: the set of free variables contained in this solution.

---


### isVariable

```
boolean isVariable(java.lang.Object obj)
```

:   Indicates if the provided object is a variable in this
    solution.

    :   **Parameters:**: `obj` - the potential variable. **Returns:**: `true` if the provided object is a variable in this solution; `false` otherwise.

---


### getSolution

```
LinearComb<V> getSolution(java.lang.Object variable)
```

:   Returns the linear combination of free variables to which the provided
    variable is equal.

    :   **Parameters:**: `variable` - a variable from this solution. **Returns:**: the linear combination of free variables to which the provided variable is equal.

---


### toMap

```
java.util.Map<V,LinearComb<V>> toMap()
```

:   Returns this solution as a map.

    :   **Returns:**: the variables of the original system of equations mapped to the linear combination of free variables to which they equal.

---


### simplify

```
LinearSystemSolution<V> simplify(java.util.Map<?,java.lang.Double> variableValues,
                                 double tolerance)
```

:   Computes and returns a simplified solution by plugging in the provided
    values. Values can be provided for both free and fixed variables.

    :   **Parameters:**: `variableValues` - variables mapped to their corresponding double precision value. Every key in the map must be a variable in this solution.: `tolerance` - the amount by which a provided or computed value is allowed to differ from any previously existing value. This should be a small positive number, like 1E-9 for example. **Returns:**: the solution resulting from plugging in the provided values.

---


### getSolutionVector

```
java.util.Map<V,java.lang.Double> getSolutionVector(java.util.Map<?,java.lang.Double> freeVariableValues)
```

:   Computes and returns a solution vector by plugging in the
    provided values for the free variables in this solution.

    :   **Parameters:**: `freeVariableValues` - free variables mapped to their corresponding double precision value. Every key in the map must be a free variable in this solution and all free variables must be present. **Returns:**: the solution vector resulting from the provided free variable values.

---


### isSolutionVector

```
boolean isSolutionVector(java.util.Map<?,java.lang.Double> variableValues,
                         double tolerance)
```

:   Indicates if the provided solution vector is consistent with
    this solution.

    :   **Parameters:**: `variableValues` - the variables of this solution mapped to their values. Every key in the map must be a variable in this solution and all variables must be present.: `tolerance` - the amount by which a provided value is allowed to differ from any computed or previously existing value. This should be a small positive number, like 1E-9 for example. **Returns:**: `true` if the provided vector is consistent with this solution; `false` otherwise.

---


### variables

```
int variables()
```

:   Returns the total number of variables in this solution. This equals
    the sum of free and fixed variables.

    :   **Returns:**: the total number of variables in this solution. **See Also:**: `freeVariables()`, `fixedVariables()`

---


### freeVariables

```
int freeVariables()
```

:   Returns the number of free variables in this solution.

    :   **Returns:**: the number of free variables in this solution.

---


### fixedVariables

```
int fixedVariables()
```

:   Returns the number of fixed variables in this solution.

    :   **Returns:**: the number of fixed variables in this solution.


---


|  |  |  |
| --- | --- | --- |
| **Overview** \| **Package** \| **Class** \| **Use** \| **Tree** \| **Deprecated** \| **Index** \| **Help** | |  |
| **PREV CLASS**   **NEXT CLASS** | **FRAMES**    **NO FRAMES**     **All Classes** |  |
| SUMMARY: NESTED \| FIELD \| CONSTR \| METHOD | DETAIL: FIELD \| CONSTR \| METHOD |  |


---
